# A synopsis of the New World species of *Drypetes* section *Drypetes* (Putranjivaceae) with asymmetrical fruits, including description of a new species

**DOI:** 10.3897/phytokeys.29.6004

**Published:** 2013-11-22

**Authors:** Geoffrey A. Levin

**Affiliations:** 1 Illinois Natural History Survey, Prairie Research Institute, University of Illinois at Urbana-Champaign, 1816 South Oak Street, Champaign, Illinois 61820

**Keywords:** Costa Rica, *Drypetes*, Mexico, Putranjivaceae, West Indies

## Abstract

A synopsis of the New World species of *Drypetes* (Putranjivaceae) with asymmetrical drupes is presented. The group consists of three species: *Drypetes alba*, with two varieties,from the West Indies, *Drypetes gentryi* from Mexico, and the newly described *Drypetes asymmetricarpa*from Costa Rica. The new species can be distinguished from both its relatives by its longer fruiting pedicels. In addition, the new species differs from *Drypetes alba* by its larger fruits, and from *Drypetes gentryi* by having shorter staminate pedicels and stigmas borne on styles (rather than sessile). Lectotypes are designated for *Drypetes alba*var. *latifolia*and *Drypetes incurva*.

## Introduction

The genus *Drypetes* Vahl (Putranjivaceae) contains about 220 species of dioecious trees and shrubs, mostly of the Old World tropics. About 17 known species are found in the Americas, with the greatest diversity in the West Indies. However the Amazonian species are poorly studied and further research undoubtedly will yield many new species. In the classification of Pax and Hoffmann (1922), the most recent comprehensive treatment available for the genus, all but three of the American species belong to the pantropical section *Drypetes* based on their pistil consisting of a single carpel [although Pax and Hoffmann named this section *Hemicyclia* (Wight & Arn.) Pax & K. Hoffm., it contains *Drypetes glauca* Vahl, the type of the genus, and therefore must be called *Drypetes*, as was pointed out by Airy Shaw (1969)]. The other three species have 2-carpellate pistils and belong to section *Oligandrae* Pax & K. Hoffm. with 3–4(–7) stamens [*Drypetes lateriflora* (Sw.) Krug & Urb.] or section *Sphragidia* (Thwaites) Pax & K. Hoffm. with 8–12(–50) stamens (*Drypetes brownii* Standl. and *Drypetes guatemalensis* Lundell); both of these sections also are pantropical. It is worth noting *Drypetes* has not been examined phylogenetically and the classification by Pax and Hoffmann (1922) may not reflect evolutionary relationships (Levin 1986).

**Figure 1.**
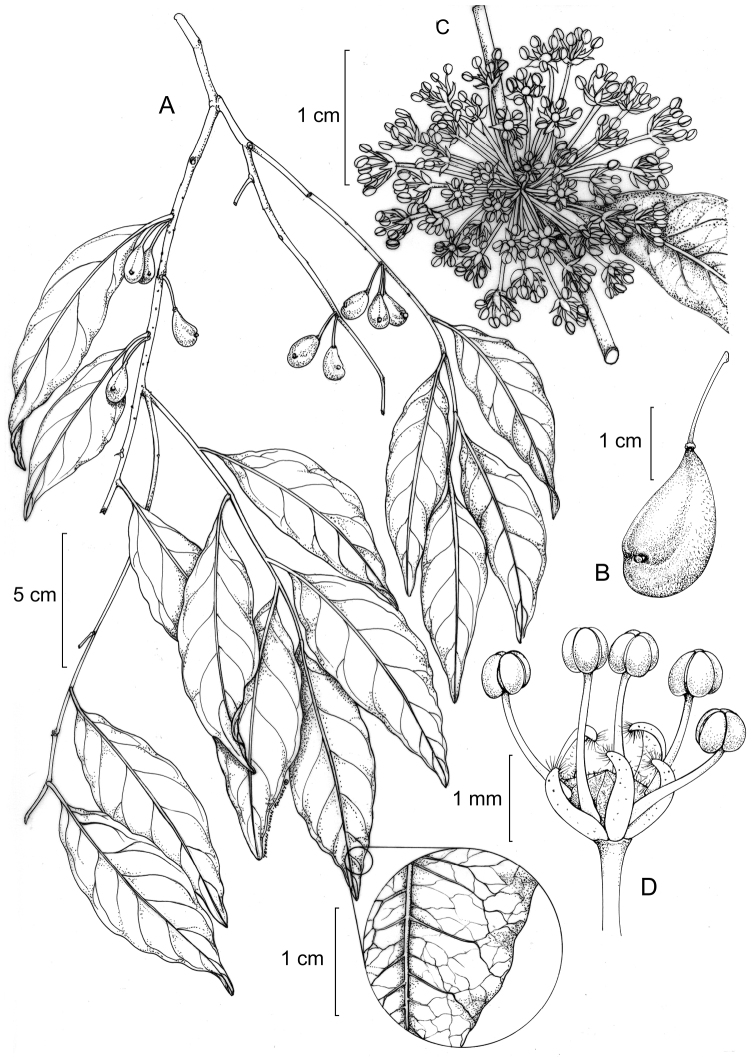
*Drypetes asymmetricarpa*. **A** Fruiting branch (detail of venation) **B** Immature fruit **C** Staminate inflorescence **D** Staminate flower. (**A–B** from *Grayum 6614*, MO; **C–D** from *Harmon 41*, MO).

Although no formal groups below the sectional level have been recognized among New World members of section *Drypetes*, there is a distinctive group of species with strongly asymmetrical drupes. In these species, the young ovary is symmetrical, as in other members of section *Drypetes*, but as the fruit develops the ovary grows faster on one side than the other, resulting in an oblique fruit apex with the stigma shifted to one side (Fig. 1B). As many as four species commonly have been recognized in this group. Here these are reduced to two species, one with two varieties, and a new species is described. The south Asian species *Drypetes gardneri* (Thwaites) Pax & K. Hoffm., *Drypetes lanceolata* (Thwaites) Pax & K. Hoffm., and *Drypetes venusta* (Wight) Pax & K. Hoffm. have somewhat similar fruits but differ significantly from the American species in foliar and floral characters and probably are not closely related.

## Methods

The study was based on the examination of specimens from the following herbaria: A, ARIZ, BM, CAS, CM, CR, DAV, F, G, G-DC, GH, GOET, HAJB, ILLS, K, LL, MEXU, MICH, MO, NY, P, SD, TEX, U, UC, UCR, and US. All cited specimens were seen by the author unless otherwise indicated. Identifications were made by comparison with the original descriptions and, when available, with the type material or photographs of types. Details of the flowers and fruits were examined under a stereoscopic microscope. All descriptions and data on flowering times, habitats, and distribution are based on the herbarium material examined. Countries in the Selected Specimens sections are listed alphabetically.

## Taxonomic treatment

### Key to the New World asymmetrical-fruited *Drypetes*

**Table:** 

1	Fruiting pedicels (8–)10–15 mm long; Costa Rica	2. *Drypetes asymmetricarpa*
–	Fruiting pedicels 4–10 mm long; Mexico and West Indies
2	Staminate pedicels 7–14 mm long; drupes 12–15 mm long; Mexico	3. *Drypetes gentryi*
–	Staminate pedicels 3–6 mm long; drupes 9–10(–13) mm long; West Indies	[1. *Drypetes alba*]
3	Stigmas sessile; Hispaniola, Puerto Rico, U.S. Virgin Islands	1a. *Drypetes alba* var. *alba*
–	Stigmas borne on style 1 mm long; Cuba, Jamaica, Lesser Antilles	1b. *Drypetes alba* var. *latifolia*

#### 1a. Drypetes alba alba

Poit., Mém. Mus. Hist. Nat. 1: 157, t. 7. 1815;

Liparena alba (Poit.) Poit. ex Baill., Étude Euphorb. 608. 1858. Type: Based on *Drypetes alba* Poit.
Drypetes sessiliflora Baill., Étude Euphorb., Atlas: 45. 1858, nom. illeg. Type: Based on *Drypetes alba* Poit.
Drypetes alba var. *genuina* Müll. Arg. in DC., Prodr. 455. 1866, nom. inval. Type: Based on *Drypetes alba* Poit.
Guatteria berteriana Spreng., Syst. Veg. (ed. 16) 2: 635. 1825. Type: Puerto Rico, s.d., *C. G. L. Bertero s.n*. (holotype: B†, isotype: TO n.v.).
Guatteria prinoides Spreng., Syst. Veg. (ed. 16) 2: 635. 1825. Type: Haiti, s.d., *C. G. L. Bertero s.n*. (holotype: B†, isotypes: MO, TO n.v.).
Drypetes alba var. *brevipes* Müll. Arg. in DC., Prodr. 455. 1866. Type: Puerto Rico, s.d., collector unidentified (holotype: G-DC).

##### Type.

[Haiti.] Île de Tortue, s.d., *A. Poiteau s.n*. (holotype: P; isotype: G-DC).

##### Distribution.

Haiti, Dominican Republic, Puerto Rico, and U.S. Virgin Islands.

##### Ecology.

Forests from sea level to 1000 m, primarily on calcareous soils, but in Puerto Rico also on serpentine-derived soils.

##### Phenology.

Flowering primarily January to April, rarely as early as November. Fruiting February to August.

##### Conservation status.

Least concern. *Drypetes alba* var. *alba* is widespread and likely secure in the Dominican Republic, Puerto Rico, and U.S. Virgin Islands. It may be vulnerable or endangered in Haiti due to extensive deforestation there; I have seen no specimens from Haiti collected later than 1929.

##### Selected specimens examined.

**HAITI.** Southeast of St. Louis du Nord, 3 Apr 1928 (♂ fl), *Leonard & Leonard 14268* (CM, K, UC, US).

**Dominican Republic.** Loma Mala, near arroyo Guayabal, Maimón, 300–500 m, 16 Feb 1974 (fr), *Liogier 21293* (F, K, MO).

**Puerto Rico.** Maricao Afuera, along Maricao River upstream from the fish hatchery, 18°10'11"N, 66°59'10"W, 460–580 m, 11 Jan 1996 (♀ fl), *Acevedo-Rodríguez et al. 7724* (K, US).

**United States Virgin Islands. Saint Croix:** Salt River bluffs, 6 Jul 1896 (♂ fl), *Ricksecker 462* (P). **Saint John:** Coral Bay Quarter, Bordeaux Mtn., east side, 11 Jan 1992 (bud), *Acevedo-Rodríguez & Siaca 4710* (MO, US). **Saint Thomas:** s.d. (fr), *Riedlé s.n*. (F, P).

#### 1b. Drypetes alba latifolia

Griseb., Nachr. Königl. Ges. Wiss. Georg-Augusts-Univ. 1: 165. 1865.

Drypetes crocea Poit. var. *latifolia* (Griseb.) Müll. Arg., in A. P. de Candolle, Prodr. 15(2): 456. 1866. Type: Based on *Drypetes alba* Poit. var. *latifolia* Griseb.
Drypetes latifolia (Griseb.) C.Wright, Anales Acad. Ci. Méd. Habana 7: 151. 1870. Type: Based on *Drypetes alba* Poit. var. *latifolia* Griseb.
Koelera ? *serrata* Maycock, Fl. Barbad. 38. 1830. Type: No specimens cited or located [according to Stafleu and Cowan (1981), Maycock probably made no herbarium; no type has ever been cited for this name].
Drypetes serrata (Maycock) Krug & Urb., Bot. Jahrb. Syst. 15: 354. 1892. Type: Based on *Koelera serrata* Maycock.
Drypetes serrulata Pax & K.Hoffm. in H.G.A.Engler, Pflanzenr., IV, 147, XV: 267. 1922, nom. superfl. Type: Based on *Koelera serrata* Maycock.
Drypetes glomerata Griseb., Abh. Königl. Ges. Wiss. Göttingen 7: 75. 1857. Type: Guadeloupe.n.d., *E. P. Duchassaing s.n*. (holotype: GOET, photo plants.jstor.org/specimen/goet006390).
Drypetes glomerata Griseb. var. *genuina* Müll.Arg. in A.P.de Candolle, Prodr. 15(2): 454. 1866, nom. inval. Type: Based on *Drypetes glomerata* Griseb.
Drypetes incurva Müll. Arg., Linnaea 32: 82. 1863. Type: Cuba, prope Havana, 1833 (fr), *R. de la Sagra 607* (lectotype, designated here: G-DC, duplicate: K).
Drypetes glomerata Griseb. var. *olivacea* Müll. Arg. in DC., Prodr. (DC.) 15(2): 454. 1866. Type: Cuba, 1860–1864, *C. Wright 1929* (holotype: G-DC, isotypes: BM, F, GH, K, MO, NY).
Drypetes serrata (Maycock) Krug & Urb.var. *olivacea* (Müll. Arg.) Krug & Urb., Bot. Jahrb. Syst. 15: 355. 1892. Type: Based on *Drypetes glomerata* Griseb. var. *olivacea* Müll. Arg.

##### Type.

Cuba, occ., 1863 (fr), *Wright 1927* (lectotype, designated here: GOET003380, photo plants.jstor.org/specimen/goet003380; duplicates: GH pro parte, K pro parte, MO pro parte, NY pro parte).

##### Distribution.

Cuba, Jamaica, and the Lesser Antilles (Antigua, Guadeloupe, Martinique, Barbados).

##### Ecology.

Forests on limestone and schist, from sea level to 1100 m.

##### Phenology.

Flowering October to March. Fruiting December to July.

##### Conservation status.

Least concern. *Drypetes alba* var. *latifolia* is widespread and probably secure in Cuba and Jamaica. Its status in the Lesser Antilles is difficult to assess because of a paucity of specimens.

##### Discussion.

The plants I include in *Drypetes alba* have been segregated into species or varieties in various ways since the middle of the 19^th^ century (Grisebach 1857; 1865; Howard 1989; León and Alain 1953; Müller 1863; 1866; Pax and Hoffmann 1922). Characters that have been used include those of the leaves (petiole length and blade color, shape, and degree of marginal serration), staminate flowers (size, pedicel length, and stamen exsertion), and pistillate flowers/fruits (pedicel length relative to fruit length, style presence/absence, and fruit size). Most of the distinctions were based on observations of the one or two specimens available to earlier workers and these disappear when more specimens are examined. For example, Grisebach (1857), Müller (1866), and León and Alain (1953) distinguished *Drypetes alba* (equivalent to var. *alba* is this treatment) from *Drypetes glomerata* or its synonym *Drypetes serrata* (here synonyms of var. *latifolia*) on the basis of the former having staminate flowers that are more than 1 mm long borne on longer pedicels and with exserted stamens in contrast to the latter having staminate flowers that are about 1 mm long borne on short pedicels and with included stamens. These differences appear to be an artifact of flower age: specimens with immature flowers, generally with the anthers indehiscent, were called *Drypetes glomerata* or *Drypetes serrata*, whereas those bearing fully mature flowers with dehiscent anthers were called *Drypetes alba*. Pax and Hoffmann (1922) separated the same taxa using petiole length: 5–8 mm for *Drypetes alba* vs. about 1 cm for *Drypetes serrulata*, the superfluous name they used for what Grisebach and Müller called *Drypetes glomerata*. In their concept, *Drypetes serrulata* is restricted to the Lesser Antilles whereas *Drypetes alba* is found throughout the Greater Antilles. Measurement of specimens shows that plants from the Lesser Antilles have slightly longer petioles (7–12 mm vs. 5–10 mm) than those from farther west, but clearly the variation is great and broadly overlapping. As I treat them, the two varieties have completely overlapping petiole lengths (6–10 mm long for var. *alba* vs. 5–12 mm for var. *latifolia*). Howard (1989) reported that *Drypetes serrata*, which he considered to be restricted to the Lesser Antilles, had larger fruits than *Drypetes alba* of the Greater Antilles, but he did not provide comparative measurements. Although mature fruits of *Drypetes alba* are rarely found on herbarium specimens, those I have seen are about 12–13 mm long throughout its range. Differences in leaf color, shape, and degree of marginal serration, alone or in combination, were used by Grisebach (1865) and Müller (1866) to describe new varieties based on single specimens, but these characters have been ignored by subsequent authors, presumably because they found, as I have, that these characters vary considerably even within individuals and certainly do not show consistent patterns.

The only character that seems consistently to differentiate *Drypetes alba* var. *alba* from var. *latifolia* is the presence of a style about 1 mm long in the former and its absence in the latter, the stigma being sessile. This character was first observed by Müller (1863) when he described *Drypetes incurva* having a sessile stigma; he later noted the same condition in *Drypetes glomerata* (Müller, 1866). I have found that all specimens from an individual island show the same condition and I have seen no intermediate specimens. Although this character is consistent geographically, in the absence of additional differences it seems too minor to support more than a varietal distinction. The distribution of the varieties is curious, with var. *latifolia* found both east and west of var. *alba*. DNA sequence data might elucidate this interesting distribution and clarify the evolutionary history of *Drypetes alba*.

Grisebach (1865) based *Drypetes alba* var. *latifolia* on *Wright 1927*. It has long been recognized that this collection, like many of Wright’s *Drypetes* collections, is a mixture of two species, in this case *Drypetes alba* and *Drypetes lateriflora* (Krug and Urban 1892; Pax and Hoffmann 1922). The material at GOET includes two sheets, both from the Grisebach Herbarium, and therefore presumably is the original material studied by Grisebach. These have the additional numbers 46 and 47 on the labels. The sheet labeled 46 (GOET 7917) consists of staminate and pistillate flowering branches of *Drypetes lateriflora* and the sheet labeled 47 (GOET 3380) consists of a fruiting specimen of *Drypetes alba* var. *latifolia*. Grisebach briefly described the leaves, staminate flowers, and fruits (“drupa”), thus he must have considered both sheets to be his new variety. In deciding which material best matches the protologue, the staminate flowers argue for GOET 7917 (*Drypetes lateriflora*)and the fruits argue for GOET 3380 (*Drypetes alba* var. *latifolia*). However Grisebach described the leaves as being subentire. The leaves of *Drypetes lateriflora* on GOET 7917 are completely entire, whereas the leaves of *Drypetes alba* var. *latifolia* on GOET 3380 are very shallowly crenulate-serrulate. The latter sheet thus better matches the protologue of D. *alba* var. *latifolia* and therefore I designate it as the lectotype.

Müller (1863) based *Drypetes incurva* on two collections, *de la Sagra 607* and *Wright 593*, pro parte [this collection number includes material of *Drypetes incurva* (= *Drypetes alba* var. *latifolia*) and *Drypetes lateriflora*]. Later, Müller (1866) cited only *de la Sagra 607* under *Drypetes incurva*, placing *Wright 593* under *Drypetes crocea* Poit., a synonym of *Drypetes lateriflora*. Based on the protologue of *Drypetes incurva*, either sheet at G-DC could be chosen as the lectotype, but because it is not a mixed collection and therefore minimizes the potential for confusion, I designate *de la Sagra 607* as the lectotype.

##### Selected specimens examined. 

**CUBA. Camagüey:** Banao, 300–500 m, Nov 1975 (♂ fl), *Alvarez et al. 28778* (HAJB). **Guantánamo:** San Antonia del Sur, Puriales de Caujeri, Sierra de Purial cerca de Arroyo, 800 m, 30 May 1982 (fr), *Bisse et al. 47259* (HAJB). **Holguín:** Sierra de Nipe, prope Río Piloto, 350 m, 16 Dec 1915 (♂ fl), *Ekman 6694* (F, K, NY, U, US). **Isla de la Juventud:** Caleta Cocodrilos, 8 Mar 1916 (fr), *Britton et al. 15305* (CM, F, NY, US). **Matanzas:** Cienega, Peninsula de Zapata, montes al norte de Sto. Tomás, 19 Apr 1977 (fr), *Bisse et al. 34469* (HAJB). **Pinar del Río:** La Guásima, Rangel, Jan 1950 (♀ fl), *Liogier 1261* (GH, US). **Sancti Spíritus:** Trinidad Mountains, Arroyo Grande, 650–750 m, 11–12 Mar 1910 (fr), *Britton &Wilson 5459* (F, NY). **Santiago de Cuba:** Bayate, 20 Feb 1917 (fr), *Ekman 8544* (F, K, LL, NY, U, US).

**Jamaica. Trelawny:** Boothe district, ca. 3 mi. north of Troy, 1600 m, 14 Mar 1955 (♀ fl, fr), *Proctor 9956* (NY, US).

**Antigua and Barbuda. Antigua:** environs de St. Jean, Dec 1902 (♂ fl), *Duss 80* (NY).

**Guadeloupe.** Marie-Galante, bois de Folle-Anse, 1896 (fr), *Duss 3628* (F, MO pro parte, NY, US pro parte).

**Martinique.** Morne Saint-Martin, pied de la montagne Pelée, 1878 (♂ fl), *Duss 50* (NY).

#### 2. Drypetes asymmetricarpa

G. A. Levin sp. nov.

urn:lsid:ipni.org:names:77134216-1

http://species-id.net/wiki/Drypetes_asymmetricarpa

Figure 1

##### Diagnosis.

Differs from the other New World *Drypetes* species withasymmetrical fruits by its longer fruiting pedicels [(8–)10–15 mm vs. 4–10 mm]; also differs from *Drypetes alba* by its larger fruits [12–18 mm vs. 9–10(–13) mm], and from *Drypetes gentryi* by having shorter staminate pedicels (5–8 mm vs. 7–14 mm) and stigmas borne on styles (vs. being sessile).

##### Type.

Costa Rica. Puntarenas: Cove at NE base of peninsula, Punta Quepos (3 km S of Puerto Quepos), 9°24'N, 84°10'W, 0 m, 8 Mar 1986 (fr), *M. H. Grayum 6614* (holotype: MO, isotypes: CR n.v., F).

##### Description.

Trees 6–20 m, to 35 cm dbh; bark with longitudinal fissures; branches brown when young, becoming gray, glabrous or sparsely minutely puberulent with spreading hairs. Leaves: stipules 0.5 × 0.5 mm, deltate, puberulent; petiole 3–10 × 0.3–1 mm, glabrous; blade elliptic to lanceolate, straight or somewhat curved, 4–12 × 1.5–4.5 cm, base asymmetrical, acute, margins entire or minutely crenulate-serrulate, often undulate, apex attenuate, surfaces glabrous, 2° veins 6–9/side. Inflorescences axillary fascicles; staminate 25–40-flowered, bracts 0.5 mm, puberulent, pedicels 5–8 × 0.2 mm, glabrous; pistillate (known only in fruit) 2–6-flowered, bracts 0.25 × 0.25 mm, deltate, puberulent, pedicels (8–)10–15 × 0.4–0.8 mm, glabrous. Staminate flowers: sepals 5–6, narrowly triangular to narrowly lanceolate, 1 × 0.3 mm, spreading and slightly incurved at apex, apex acute, margins ciliate, abaxial surface glabrous except puberulent at apex, adaxial surface densely to sparsely puberulent; stamens 5(–6), irregularly alternate and opposite sepals, filaments 1.5–2 mm × 0.1 mm, glabrous, anthers 0.4–0.5 × 0.4–0.5 mm, glabrous, latrorse; disc lobed between stamens, densely puberulent. Pistillate flowers unknown, but remnant sepals (below fruits) ovate-elliptic, 1.5 × 0.6 mm, apex acute and slightly incurved, abaxial surface glabrous, adaxial surface densely puberulent; disc annular, densely puberulent; ovary unknown; style becoming subapical during fruit development, 0.5 mm; stigma subreniform, 0.5 × 1 mm, glabrous. Drupes (immature) green, 1-carpellate, ovoid-globose, 12–18 × 7–10 × 6–8 mm, apex strongly asymmetrical, sparsely to densely puberulent with very short hairs (0.1 mm). Seed 1.

##### Etymology.

The specific epithet refers to the strongly asymmetrical drupes, which are unique among Central American *Drypetes*.

##### Distribution.

Known only from Costa Rica, where it is found from the north central part of the country to the central west coast. It may also be expected in extreme southern Nicaragua.

##### Ecology.

Forests at elevations from sea level to 750 m.

##### Phenology.

Flowering January (possibly longer, but only a single flowering specimen known). Fruiting March to June (possibly longer as only immature fruits are known).

##### Conservation status.

Probably of Least Concern. The range of *Drypetes asymmetricarpa* spans at least 200 km. Although its habitat is highly fragmented, the species is found in Manuel Antonio National Park and near both Guanacaste and Rincón de la Vieja national parks.

##### Discussion.

*Drypetes asymmetricarpa* was listed by Burger and Huft (1995) as *Drypetes* sp. aff. *Drypetes alba* and by González (2010) as *Drypetes* sp. 1 and sp. 2.

All the pistillate specimens studied have immature fruits, so their full size and color at maturity are unknown. The label on *Hammel & Trainer 17046*, with fruits 9–11 × 7–8 × 6–7 mm, says “fruits ca. 1/3 full size,” but based on my experience with other species I suspect this overestimates the mature size.

##### Specimens examined.

**COSTA RICA. Alajuela:** Cantón de Upala, Distrito Dos Ríos, 7.5 km NE of town, between La Jabalina and the Río Cucaracho, 10°56'N, 85°19'W, 325 m, 4 Apr 1988 (fr), *Herrera 1693* (F, ILLS, MO); **Puntarenas:** Cantón de Puntarenas, Distrito Monteverde, San Luis, finca de Chepe Rojas, al oeste del pueblo, 10°16'N, 84°50'W, 750 m, 24 Jun 1988 (fr), *Bello et al. 35* (F, ILLS, MO), *Bello et al. 58* (F, ILLS, MO); Monteverde area from Santa Elena to San Luis, 10°16'N, 84°50'W, 700 m, 16 Jun 1988 (fr), *Hammel & Trainer 17046* (F, ILLS, MO); Parque Nacional Manuel Antonio, Playa Espadilla Sur. 9°24'N, 84°10'W, 1–100 m. 2 Jan 1990 (♂ fl), *Harmon 41* (MO).

#### 3. Drypetes gentryi

Monach., Phytologia 3: 32. 1948, as “gentryii”

http://species-id.net/wiki/Drypetes_gentryi

##### Type.

Mexico. Sinaloa: Capadero, Sierra Tacuichamona, rocky canyon under basaltic rim, 3500 ft., 13 Feb 1940 (fr), *H. S. Gentry 5597* (holotype: NY, isotypes: ARIZ, MICH, MO).

##### Distribution.

Western Mexico, in the Sierra Madre Occidental from near 27° N in Chihuahua and Sonora to about 19° N in Colima.

##### Ecology.

Tropical deciduous forests at about 100–1100m.

##### Phenology.

Flowering December–February. Fruiting December–June.

##### Conservation status.

Least Concern. *Drypetes gentryi* is widespread in the lower elevations of the Sierra Madre Occidental.

##### Discussion.

When Monachino (1948) described *Drypetes gentryi*, he examined only a single specimen. No other descriptions of the species have been published, so I provide here an expanded description:

Trees 8–25 m, often with multiple trunks from near base, to 20–100 cm dbh; bark scaled and with longitudinal fissures; branches brown when young, becoming gray, minutely puberulent with spreading hairs, becoming glabrous. Leaves: stipules 0.5–0.6 × 0.7–1 mm, deltate, puberulent; petiole 6–12 × 0.7–1 mm, puberulent with spreading hairs or glabrous; blade elliptic to lanceolate, straight or somewhat curved, 4–15 × 1.5–4 cm, base asymmetrical, acute to narrowly obtuse, margins subentire to crenulate-serrulate, often undulate, apex attenuate, surfaces glabrous or very sparsely pubescent with appressed hairs especially near base, 2° veins 6–9/side. Inflorescences axillary fascicles; staminate 20–40-flowered, bracts 0.5 × 0.5 mm, deltate, puberulent, pedicels 7–14 × 0.2 mm, glabrous; pistillate 1–6-flowered, bracts 0.5 x0.5 mm, deltate, puberulent, pedicels 3–10 × 0.4–0.5 mm, puberulent when young, becoming glabrous. Staminate flowers: sepals 5(–6), linear to narrowly triangular, 1.2 × 0.4 mm, spreading and slightly incurved at apex, apex bluntly acute, margins ciliate, abaxial surface glabrous except puberulent at apex, adaxial surface puberulent; stamens 5(–6), mostly opposite sepals, filaments 1.6–2.2 mm × 0.1 mm, glabrous, anthers 0.8–1 × 0.5–0.6 mm, glabrous, latrorse; disc lobed between stamens, densely puberulent. Pistillate flowers: sepals 5, narrowly triangular to linear, 1–1.2 × 0.3–0.4 mm, spreading, entire, apex bluntly acute, abaxial surface glabrous to sparsely puberulent but densely puberulent at apex, adaxial surface densely puberulent; disc annular, densely puberulent; ovary densely puberulent; style absent; stigma apical at anthesis, becoming subapical during fruit development, subreniform, 0.8 × 1.2 mm, glabrous. Drupes (immature) green, 1-carpellate, obovoid, 12–15 × 7–9 × 6–8 mm, apex strongly asymmetrical, densely puberulent with very short hairs (0.1 mm). Seed 1.

The mature fruits are described as white (*Bye 6066*) or yellow (*Bye et al. 12847*), with the mesocarp juicy and both sweet and astringent (*Bye et al. 12847*). Spanish vernacular names include *cortopico* (*Gentry 5597*), *palo masiso* (*Bye 9707*), and *tempisque* (*Bye 3401*, *Bye et al. 12847*); in Tarahumara it is called *bapible* (*Bye 3401*) or *kafe* (*Bye et al. 12847*), and in Guarijio *joyarí* (*Felger et al. 94-56*).

##### Selected specimens examined.

**MEXICO. Chihuahua:** Mpio. Batopilas, north side of Barranca de Batopilas, along arroyo Samachique between Rio Batopilas and Tarahuamara village of Wimivo, 27°09'N, 107°38'W, 900–1000 m, 30 May 1980 (fr), *Bye 9707* (ARIZ, DAV, F, GH, ILLS, MEXU, MICH, MO, NY, SD, TEX, UCR, US); **Colima:** canyon near Rio Marabasco (Cihuatlan) bridge on road to Chacala, north of Santiago, 19°17'N, 104°19'W, 200–250 m, 21 Jan 1988 (fr), *Levin & Dice 1975* (MO, SD); **Jalisco:** canyon east of Highway 200 ca. 2 km east-southeast of Boca de Tomatlan, at bridge, 20°03'N, 105°18'W, 100–200 m, 25 Jan 1988 (♂), *Levin & Dice 2001* (MO, SD); **Sonora:** Arroyo Gochico ca. 8 km E of San Bernardo, 27°02'04"N, 108°04'07"W, 300 m, 31 Jan 1988 (♀ fl, fr) *Levin et al. 2015* (MO, SD).

## Supplementary Material

XML Treatment for Drypetes alba alba

XML Treatment for Drypetes alba latifolia

XML Treatment for Drypetes asymmetricarpa

XML Treatment for Drypetes gentryi

## Index to numbered collections

Abbott, W.L. 2571, 2932 (alba var. alba)

Acevedo-Rodríguez, P. 4710, 4781, 7724 (alba var. alba)

Alvarez, A. 28778, 45576 (alba var. latifolia)

Areces, A. 25812 (alba var. latifolia)

Bello, E. 35, 58 (asymmetricarpa)

Bisse, J. 16899, 22625, 26478, 34469, 46192, 47203, 47259, 53290 (alba var. latifolia)

Britton, E.G. 5154 (alba var. alba)

Britton, N.L. 2457, 2748, 4128, 4208 (alba var. alba); 5459 (alba var. latifolia); 8104, 9676 (alba var. alba); 15305 (alba var. latifolia)

Bye, R.A. 3401, 3557, 3558, 3958, 6066, 9707, 12847 (gentryi)

Duke, J.A. 7350 (alba var. alba)

Duss, A. 50, 80 (alba var. latifolia); 3628 (alba var. latifolia and Drypetes glauca Vahl); 4142 (alba var. latifolia)

Eggers, H.F.A. 2612 (alba var. alba)

Ekman, E.L. 4146 (alba var. latifolia); 4179 (alba var. alba); 4543, 4568, 4838, 5006 (alba var. latifolia); 5528 (alba var. alba); 6694, 8544, 9764, 9913 (alba var. latifolia); 12160 (alba var. alba); 12462, 18213 (alba var. latifolia); H.3512, H.3870, H.4178, H.4179, H.5134, H.5528, H.6061, H.9614, H.12160 (alba var. alba)

Felger, R.S. 94-56, 97-118 (gentryi)

Gentry, A.H. 50716 (alba var. alba)

Gentry, H.S. 3618, 5597 (gentryi)

Grayum, M.H. 6614 (asymmetricarpa)

Haenke, T. 1605 (gentryi)

Hammel, B.E. 17046 (asymmetricarpa)

Harmon, W.E. (asymmetricarpa)

Harris, W. 7098 (alba var. latifolia)

Herrera, G. 1693 (asymmetricarpa)

Holdridge, L.R. 149 (alba var. alba)

Jack, J.G. 7809 (alba var. latifolia)

Jiménez Almonte, J. de J. 1981, 4360, 5058 (alba var. alba)

León, Bro. 6081, 11800 (alba var. latifolia)

Leonard, E.C. 11589, 14257, 14268, 15686 (alba var. alba)

Lesueur, D.H. 1419 (gentryi)

Levin, G.A. 1973, 1975, 2001, 2014, 2015 (gentryi)

Liogier, A.H. 1017, 1261, 1275, 2997, 5719 (alba var. latifolia); 10466, 10714, 10779, 14325, 15661, 20858, 21293, 21600, 22628, 24490, 24537, 25846, 26463, 26835 (alba var. alba)

Little, E.L., Jr. 13297, 13366 (alba var. alba)

Luna, A. 15 (alba var. latifolia)

McVaugh, R. 1647, 25404 (gentryi)

Pérez J., L.A. 413 (gentryi)

Proctor, G.R. 9956 (alba var. latifolia)

Ricksecker, A.E. 462 (alba var. alba)

Sagra, R. de la 607 (alba var. latifolia)

Sanders, A.C. 1096 (gentryi)

Schiffino, J. 15 (alba var. alba)

Shafer, J.A. 3693, 4403 (alba var. latifolia)

Sintenis, P.E.E. 1502 (alba var. alba)

Stehlé, H. 416 (alba var. latifolia)

Valeur, E.J. 837 (alba var. alba)

Van Devender, T.R. 93-1381 (gentryi)

Wilson, P. 9287 (alba var. latifolia)

Wright, C. 498, 501 (alba var. alba and Drypetes lateriflora (Sw.) Krug & Urb.); 593 (alba var. latifolia and D. lateriflora); 1112 (alba var. latifolia); 1927 (alba var. latifolia and D. lateriflora); 1928, 1929, 1929, 593b (alba var. latifolia)

Zanoni, T.A. 36401 (alba var. alba)
